# Supplementary material for: The mini-Oxford cognitive screen (Mini-OCS): A very brief cognitive screen for use in chronic stroke
Source: Eur Stroke J. 2025 Jul 27:23969873251358811. Online ahead of print. doi: 10.1177/23969873251358811 (PMC12301230; doi:10.1177/23969873251358811)
Supplement: sj-docx-1-eso-10.1177_23969873251358811 – Supplemental material for The mini-Oxford cognitive screen (Mini-OCS): A very brief cognitive screen for use in chronic stroke [file sj-docx-1-eso-10.1177_23969873251358811.docx]

**Supplementary materials – Mini - Oxford Cognitive Screen (Mini-OCS)**

**Development of the Mini-Oxford Cognitive Screen (Mini-OCS)**

Existing full OCS data for 464 participants who completed the OCS at least 6 months post stroke were analysed to determine the possibility of a short form. The data were part of the OCS-Recovery (Milosevich et al., 2023) and OCS-Care (Demeyere et al., 2019) studies.

Additional theoretical choices were made to adapt the short form suitable for use in chronic stroke. We started with a core set of essential subtasks to include from the OCS, which covered the cognitive domains. At the most minimal level, we aimed to include: 1) orientation (core memory domain), 2) sentence reading (core language domain), 3) broken hearts cancellation test (core attention), 4) gesture imitation (core praxis) and 5) trail making test (only the mixed trails; core executive function), to adhere to national and international guidelines for cognitive screening in stroke (Intercollegiate Stroke Working Party, 2023; National Institute for Health and Care Excellence, 2023; Quinn et al., 2021).

Next, we considered alternative, extended, minimal datasets with further subtasks included such as the recall tasks of the sentence and number writing/cancellation. We used classical test theory and IRT modelling to create a short-form OCS, from which we selected the best model which fit statistical, theoretical, and practical criteria. Confirmatory Factor Analysis (CFA) was used to assess unidimensionality for subsequent analyses. Item response theory methods were employed to establish discrimination and difficulty of each OCS subtask, which enabled better selection of informative items. The finalised Mini-OCS test was decided following iterative piloting and minor refinements.

***CFA and IRT modelling***

Existing chronic OCS data (12 subtasks, each a binary impairment variable) were modelled using unidimensional CFA, with a factor loading cut-off of >.39 for inclusion in a factor (Li et al., 2019; Lyden et al., 2004). Subsequently, a 2-parameter logistic IRT model (discrimination and difficulty) was fit. Two theta values (measurement of the underlying construct of the OCS tasks) were estimated for each participant: one based on the full form with all 12 subtasks, and one based on the proposed short-form with the selected tasks covering the different cognitive domains.

**Development: Results**

**CFA and IRT modelling**

Complete data for all subtasks in the OCS was available for 464 stroke survivors. The unidimensional CFA model showed a good model fit (CFI: 0.981, RMSEA: 0.024). All factor loadings were significant and salient (above 0.40). No signs of local dependency were found. All subtasks of the OCS had discriminations above 1, and all subtasks had relatively high difficulty on average (mean difficulty= 1.95; *SD*= .44; range= 1.19 to 2.73). Using different numbers of subtests with the highest discriminations (the discrimination parameter indicates the overall information one can obtain from each subtest), an increasing correlation between the estimated theta and the full-scale theta was observed with increasing numbers of subtests included. With three subtests, the correlation was .72, with six subtests .85. Assuming this unidimensional structure, the results suggest that we could shorten the test by half (i.e. from 12 to six subtests) without losing much precision. Several individual models were found with moderate to high correlations between the theta values, see next for additional information for each model:

**IRT models of data**

- Model 1 (r=.93) consisting of: orientation, number calculation, verbal memory, praxis, broken hearts, trails, and sentence reading
- Model 2 (r=.91) consisting of: orientation, sentence reading, broken hearts, number calculation, trails, and verbal memory
- Model 3 (r=.90) consisting of: orientation, sentence reading, broken hearts, trails, and verbal memory
- Model 4(r=.85) consisting of: orientation, sentence reading, broken hearts, number calculations, and trails
- Model 5 (r=.83) consisting of orientation, sentence reading, broken hearts, and trials.

The final model for the Mini-OCS (i.e., model 1) was chosen based on the highest theta correlation achieved with the short form. The final Mini-OCS is thus composed of the following 9 subtasks; 1) orientation, 2) calculation, 3) word encoding and 4) recall, 5) meaningless gesture imitation, 6) shortened broken hearts test, 7) mixed trails, 8) delayed word recall, and 9) sentence reading. Justification for test selection is detailed next.

**Practical and theoretical considerations for Mini-OCS**

The test-materials needed were reduced by incorporating the instructions for the cancellation and trail making tasks onto the test sheet for reference, rather than use a separate practice. Default multiple-choice options were removed, and instead a separate multiple-choice option is to be used where communication difficulties are present. To further reduce testing time, a shorter version of the broken hearts test with 30 rather than 50 hearts is included (version with similar crowding in vertically centred subsection of the page, as developed and normed for OxVPS (Vancleef et al., 2025). In addition, we employed a higher-contrast version to allow an approximate visual acuity of 1.0 LogMAR equivalent at 30cm distance, to aid those with poorer visual acuity or lack of availability of their reading glasses. This lower visual acuity adaptation of the OCS broken hearts task was developed with orthoptic input as part of a separate project (Hepworth et al., 2025).

To increase the sensitivity of the Mini-OCS to milder domain-general impairments at a chronic stage (where the original OCS is less sensitive to memory in particular (Webb et al., 2022), more sensitive subtasks from OCS-Plus were chosen for Word Recall and Trail making (Demeyere et al., 2021)). Similarly, for the number calculations, we varied the specific OCS calculations to be different in case the participant recalled the original OCS, and to be slightly more difficult in the Mini-OCS.

Finally, we considered the order of tasks: This included prioritising the encoding subtasks at the start, with filler tasks, and then delayed recall as one of the last tasks. We aimed to save time and resources by having only the minimum additional sheets of paper. Four pages per patient are required for the Mini-OCS: 1) examiner form, 2) mixed trails practice sheet, 3) the mixed trails test sheet, and 4) shortened broken hearts cancellation. In addition, a reusable test booklet contains a mixed trails demonstration page, large print sentence reading task page along with multiple-choice questions and answers for those with expressive speech limitations.

**Iterative process of refinement for Mini-OCS**

Version 1.1 was made of the Mini-OCS, based on model 1 (r=.93) which included the following subtasks in order of appearance: 1) orientation (core memory), 2) number calculations (core number), 3) sentence reading (core language), 4) word encoding 1 and 2, 5) shortened broken hearts test (core visual spatial attention), 6) mixed trails test (core executive function), 7) gesture imitation (core praxis), and 8) verbal recognition (delayed recall). The calculations were chosen to be different from the OCS to avoid practice effects, and to increase challenge slightly. For instance, the calculations became 5+4, 37+9, 9-4, and 35-18. The second calculation was changed from the original OCS to appear harder but not require much more mathematical calculation, such that the addition of 9 is as hard for 7 as it is for 37, but the increase in magnitude from 7 to 37 may make participants think it is a harder calculation than it is. Version 1.1 had the original OCS-Plus mixed trails stimuli.

Version 1.1 was administered to five stroke survivors, but was found to not prioritise core deficits which might expect to see at 6-months based on our research (Milosevich et al., 2023), and that sentence reading was only included early in the test as it was part of the verbal recall task in the OCS, but it is not in the Mini-OCS. As such, in version 1.2 we moved sentence reading to be the very last subtask, such that in order the Mini-OCS then had the following tasks which remained for all further iterations: 1) orientation, 2) number calculations (5+4; 37+9; 9-4; 35-18), 3) word encoding 1 and 2, 4) gesture imitation, 5) shortened broken hearts, 6) mixed trails, 7) delayed verbal recall, and 8) sentence reading. Version 1.2 was administered to 17 stroke survivors. Version 1.2.1 was created where the trail making test was altered to have high contrast similar to the shortened broken hearts test.

We found that healthy control participants were not completing the mixed trails well, with lower scores than expected based on the OCS-Plus normative data. Whilst we collected no formal qualitative data, during the mini-OCS refinement, we did pilot the tests and incorporated feedback. For example, we noticed that several people struggled without a practice and demonstration of the Mini-OCS trail making task, as was present in the OCS-Plus version. Whilst participants did not make comments to explicitly compare the Mini-OCS and OCS-Plus, these observations and comments led us to update the subtest to have at least once practice.This alteration formed Version 1.3. Version 1.3 was run with an additional five participants before an error was noticed in the reformatted trail making test. Correction of this error, where connection 14 was not possible, resulted in version 1.3.1. Version 1.3.1 was run with 39 stroke survivors.

Further iterations involved the number calculation test, whereby, the first three calculations all involved or were answered by the number 9. We changed the third calculation from 9-4 to 7-3 instead to avoid the number 9. This change was Version 1.4 and ran with seven stroke survivors. After consultation with collaborators on the use of the number seven, we altered the seven to be eight, so that the final calculation change was changed from 7-3 to 8-3 (Version 1.4.1). Version 1.4.1 was run with 121 participants. The final MINI-OCS version was 1.4.2, which included a small practice sheet for the trails task due to low scoring of healthy controls on the mixed trails, only added later as part of version 1.4.1). This involved two squares and two circles and a total of four connections for the respondent to complete with feedback presented once by the assessor and 1 further attempt allowed before progression to the final test sheet. Version 1.4.2 was administered to 40 participants.

**Psychometric properties of Mini-OCS**

***Reliability***

There were 32 participants who were retested on the Mini-OCS (healthy ageing n = 23, and stroke n = 9) (Mean days between test-retest = 268.62, *SD*=69.01, range = 128 to 438). We used ANCOVA to covary for change in MoCA score, to account for change in global cognition between time points. See Table S1 for summary of group comparisons.

**Table S1**. Comparison between original and retested Mini-OCS subtask performance from a mixed sample of 23 healthy controls and 9 consecutively sampled stroke survivors.

| Mini-OCS Measure | Original mean | Retest mean | ANCOVA | % matched scores |
| --- | --- | --- | --- | --- |
| Orientation | 3.90 | 3.97 | F(1,1) = 1.89, p>.05, partial eta-squared = 0.22 | 91.30 |
| Number calculations | 3.65 | 3.63 | F(1,1) = 0.17, p>.05, partial eta-squared = 0.28 | 65.22 |
| Immediate recall 1 | 4.42 | 4.43 | F(1,1) = 0.03, p>.05, partial eta-squared = 0.19 | 73.91 |
| Immediate recall 2 | 4.87 | 4.70 | F(1,1) = 0.92, p>.05, partial eta-squared = 0.27 | 82.61 |
| Meaningless gesture imitation (praxis) | 1.84 | 1.63 | F(1,1) = 1.83, p>.05, partial eta-squared = 0.46 | 78.26 |
| Broken hearts total | 27.58 | 28.90 | F(1,1) = 0.39, p>.05, partial eta-squared = 0.41 | 39.13 |
| Broken hearts allocentric neglect | -0.03 | -0.03 | F(1,1) = 0.19, p>.05, partial eta-squared = 0.18 | 91.30 |
| Broken hearts egocentric neglect | 0.23 | 0.13 | F(1,1) = 0.58, p>.05, partial eta-squared = 0.22 | 47.83 |
| Executive function | 10.90 | 11.13 | F(1,1) = 0.01, p>.05, partial eta-squared = 0.4 | 47.83 |
| Delayed recall | 3.77 | 3.63 | F(1,1) = 0.24, p>.05, partial eta-squared = 0.35 | 43.48 |
| Sentence reading | 14.52 | 14.27 | F(1,1) = 0, p>.05, partial eta-squared = 0.36 | 60.87 |
| Time | 465.33 | 447.50 | F(1,1) = 3.71, p>.05, partial eta-squared = 0.45 | 21.74 |
| Mini-OCS total score | -0.09 | -0.09 | F(1,1) = 0, p>.05, partial eta-squared = 0.34 | 0 |

***Construct Validity***

Next, we found that most OCS GP subtasks were significantly related to the construct and format matched neuropsychology battery subtasks. See Table S2 which details the correlations between each OCS GP subtask and comparator task. Further, there was no significance relationships observed in the discriminant validity analyses.

**Table S2**. Convergence and discrimination of Mini-OCS subtasks compared to independent neuropsychological subtasks, including data from a mixed sample of healthy controls and stroke survivors.

| Mini-OCS task | Convergent test | Convergent Correlation | Discriminant test | Discriminant Correlation |
| --- | --- | --- | --- | --- |
| Orientation | MoCA Orientation | r(227)=.23, p< .001** | OCS Praxis | r(172)=.26, p< .001** |
| Orientation | OCS Orientation | r(173)=.45, p< .001** | OCS Praxis | r(172)=.26, p< .001** |
| Number calculations | CAT number calculations | r(170)=.31, p< .001** | OCS Praxis | r(172)=.19, p=.011* |
| Number calculations | OCS number calculations | r(172)=.16, p=.03* | OCS Praxis | r(172)=.19, p=.011* |
| Immediate recall 1 | MoCA immediate recall 1 | r(227)=.43, p< .001** | OCS Praxis | r(172)=.22, p=.003* |
| Immediate recall 2 | MoCA immediate recall 2 | r(227)=.54, p< .001** | OCS Praxis | r(172)=.31, p< .001** |
| Meaningless gesture imitation (praxis) | BDAE praxis use of objects | r(171)=.21, p=.006* | OCS Broken Hearts | r(172)=.14, p=.068 |
| Meaningless gesture imitation (praxis) | OCS praxis | r(172)=.31, p< .001** | OCS Broken Hearts | r(172)=.14, p=.068 |
| Broken hearts total | BIT star cancellation total | r(171)=.23, p=.002* | OCS Orientation | r(173)=.19, p=.013* |
| Broken hearts egocentric neglect | BIT star cancellation egocentric neglect | r(171)=.08, p=.322 | OCS Orientation | r(173)=0.05, p=.471 |
| Broken hearts total | OCS broken hearts total correct | r(172)=.39, p< .001** | OCS Orientation | r(173)=.19, p=.013* |
| Broken hearts egocentric neglect | OCS broken hearts egocentric neglect | r(172)=.06, p=.423 | OCS Orientation | r(173)=0.05, p=.471 |
| Broken hearts allocentric neglect | OCS broken hearts allocentric neglect | r(172)=0.08, p=.288 | OCS Orientation | r(173)=.13, p=.089 |
| Executive function | CLQT mixed symbol trails accuracy | r(171)=.43, p< .001** | OCS Orientation | r(173)=.16, p=.032* |
| Executive function | OCS mixed trails accuracy | r(170)=.38, p< .001** | OCS Orientation | r(173)=.16, p=.032* |
| Delayed recall | MoCA free recall | r(227)=.52, p< .001** | OCS Praxis | r(172)=.15, p=.049* |
| Delayed recall | OCS free recall | r(172)=.43, p< .001** | OCS Praxis | r(172)=.15, p=.049* |
| Sentence reading | BDAE sentence reading | r(171)=.45, p< .001** | OCS Praxis | r(172)=.07, p=.328 |
| Sentence reading | OCS sentence reading | r(172)=.38, p< .001** | OCS Praxis | r(172)=.07, p=.328 |
| Mini-OCS Time | MoCA time taken | r(221)=.57, p< .001** |  |  |
| Mini-OCS total score | MoCA total score | r(227)=.31, p< .001** |  |  |

*Note.* MoCA refers to the Montreal Cognitive Assessment. OCS refers to Oxford Cognitive Screen. CAT refers to the Comprehensive Aphasia Battery. CLQT refers to the Cognitive Linguistic Quick Test. BDAE refers to the Boston Diagnostic Aphasia Examination.

***Associations with demographics***

**Table S3.** Associations between scores on the Mini-Oxford Cognitive Screen (Mini-OCS) and age and education in years.

| Mini-OCS task | Age | Education |
| --- | --- | --- |
| Orientation | -0.19 (p=0.002**) | 0.16 (p=0.16) |
| Number calculations | -0.09 (p=0.167) | 0.09 (p=0.09) |
| Immediate recall 1 | -0.15 (p=0.018) | 0 (p<.001**) |
| Immediate recall 2 | -0.19 (p=0.002*) | 0 (p<.001**) |
| Meaningless gesture imitation (praxis) | -0.08 (p=0.196) | 0.13 (p=0.13) |
| Broken hearts total | -0.17 (p=0.007*) | 0.11 (p=0.11) |
| Broken hearts allocentric neglect | 0.03 (p=0.646) | -0.16 (p=-0.16) |
| Broken hearts egocentric neglect | 0.15 (p=0.021) | 0 (p=>0.05) |
| Executive function | -0.24 (p<.001**) | 0.17 (p=0.17) |
| Delayed recall | -0.21 (p=0.001**) | 0.16 (p=0.16) |
| Sentence reading | 0.08 (p=0.215) | 0.16 (p=0.16) |
| Time | 0.22 (p<.001**) | -0.1 (p=-0.10) |
| Mini-OCS total score | -0.03 (p>.05) | 0 (p=<.001) |

*Note.* ‘**’ refers to significance below alpha corrected level of .05/13 = .003, and ‘*’ refers to significance below .05.

**References**

Demeyere, N., Haupt, M., Webb, S. S., Strobel, L., Milosevich, E., Moore, M. J., Wright, H., Finke, K., & Duta, M. (2021). Introducing the tablet-based Oxford Cognitive Screen-Plus (OCS-Plus) as an assessment tool for subtle cognitive impairments. *Scientific Reports*, *11*(8000). https://doi.org/10.1038/s41598-021-87287-8

Demeyere, N., Sun, S., Milosevich, E., & Vancleef, K. (2019). Post-stroke cognition with the Oxford Cognitive Screen vs Montreal Cognitive Assessment: A multi-site randomized controlled study (OCS-CARE). *AMRC Open Research*, *1*(12), 12.

Hepworth, L. R., Demeyere, N., Helliwell, B., Ashall-Lee, L., Liptrot, M., McCullough, E., & Wright, L. (2025). Evaluation of an adaptation to the Oxford Cognitive Screen for reduced visual acuity: A cohort study. *Topics in Stroke Rehabilitation*, 1–7. https://doi.org/10.1080/10749357.2025.2501005

Intercollegiate Stroke Working Party. (2023). *National Clinical Guideline for Stroke for the UK and Ireland*. Intercollegiate Stroke Working Party. www.strokeguideline.org

Li, J., Li, Y., Li, P., & Ye, M. (2019). Early Symptom Measurement of Post-Stroke Depression: Development and validation of a new short version. *Journal of Advanced Nursing*, *75*(2), 482–493. https://doi.org/10.1111/jan.13885

Lyden, P., Claesson, L., Havstad, S., Ashwood, T., & Lu, M. (2004). Factor Analysis of the National Institutes of Health Stroke Scale in Patients With Large Strokes. *Archives of Neurology*, *61*(11), 1677–1680. https://doi.org/10.1001/archneur.61.11.1677

Milosevich, E. T., Moore, M. J., Pendlebury, S. T., & Demeyere, N. (2023). Domain-specific cognitive impairment 6 months after stroke: The value of early cognitive screening. *medRxiv*, 2023–06.

National Institute for Health and Care Excellence. (2023). *Stroke rehabilitation in adults: Clinical Guideline* (Guideline No. NG236). www.nice.org.uk/guidance/ng236

Quinn, T. J., Richard, E., Teuschl, Y., Gattringer, T., Hafdi, M., O’Brien, J. T., Merriman, N., Gillebert, C., Huyglier, H., Verdelho, A., Schmidt, R., Ghaziani, E., Forchammer, H., Pendlebury, S. T., Bruffaerts, R., Mijajlovic, M., Drozdowska, B. A., Ball, E., & Markus, H. S. (2021). European Stroke Organisation and European Academy of Neurology joint guidelines on post-stroke cognitive impairment. *European Stroke Journal*, 23969873211042192. https://doi.org/10.1177/23969873211042192

Vancleef, K., Castellani, R., Shorthose, R., Guo, C., Cai, M. F., Guazzo, F., & Demeyere, N. (2025). The Oxford Visual Perception Screen: Development and normative data of a standardised assessment for visual perception difficulties. *Clinical Rehabilitation*, 02692155251315606. https://doi.org/10.1177/02692155251315606

Webb, S. S., Hobden, G., Roberts, R., Chiu, E. G., King, S., & Demeyere, N. (2022). Validation of the UK English Oxford cognitive screen-plus in sub-acute and chronic stroke survivors. *European Stroke Journal*, 23969873221119940. https://doi.org/10.1177/23969873221119940
